# Supplementary material for: Routine Postoperative Antibiotic Prophylaxis Offers No Benefit after Hepatectomy—A Systematic Review and Meta-Analysis
Source: Antibiotics (Basel). 2022 May 12;11(5):649. doi: 10.3390/antibiotics11050649 (PMC9138010; doi:10.3390/antibiotics11050649)
Supplement: Supplementary file 1 [file antibiotics-11-00649-s001.zip › Supplementary Table S1.pdf]

Supplementary Materials

Table S1. Search strategy.

Database	Search strategz\y
MEDLINE	("anti bacterial agents"[Pharmacological Action] OR "anti bacterial agents"[MeSH Terms] OR ("anti bacterial"[All Fields] AND "agents"[All Fields]) OR "anti bacterial agents"[All Fields] OR "antibiotic"[All Fields] OR "antibiotics"[All Fields] OR "antibiotic s"[All Fields] OR "antibiotical"[All Fields] OR ("anti bacterial agents"[Pharmacological Action] OR "anti bacterial agents"[MeSH Terms] OR ("anti bacterial"[All Fields] AND "agents"[All Fields]) OR "anti bacterial agents"[All Fields] OR "antibiotic"[All Fields] OR "antibiotics"[All Fields] OR "antibiotic s"[All Fields] OR "antibiotical"[All Fields]) OR ("anti bacterial agents"[Pharmacological Action] OR "anti bacterial agents"[MeSH Terms] OR ("anti bacterial"[All Fields] AND "agents"[All Fields]) OR "anti bacterial agents"[All Fields] OR "antibacterial"[All Fields] OR "antibacterials"[All Fields] OR "antibacterially"[All Fields]) OR ("anti infective agents"[Pharmacological Action] OR "anti infective agents"[MeSH Terms] OR ("anti infective"[All Fields] AND "agents"[All Fields]) OR "anti infective agents"[All Fields] OR "antimicrobial"[All Fields] OR "antimicrobials"[All Fields] OR "antimicrobially"[All Fields])) AND ("hepatectomy"[MeSH Terms] OR "hepatectomy"[All Fields] OR "hepatectomies"[All Fields] OR "liver resection"[All Fields] OR "hepatic resection"[All Fields])
