# Supplementary figures and images for: Routine Postoperative Antibiotic Prophylaxis Offers No Benefit after Hepatectomy—A Systematic Review and Meta-Analysis
Source: Antibiotics (Basel). 2022 May 12;11(5):649. doi: 10.3390/antibiotics11050649 (PMC9138010; doi:10.3390/antibiotics11050649)

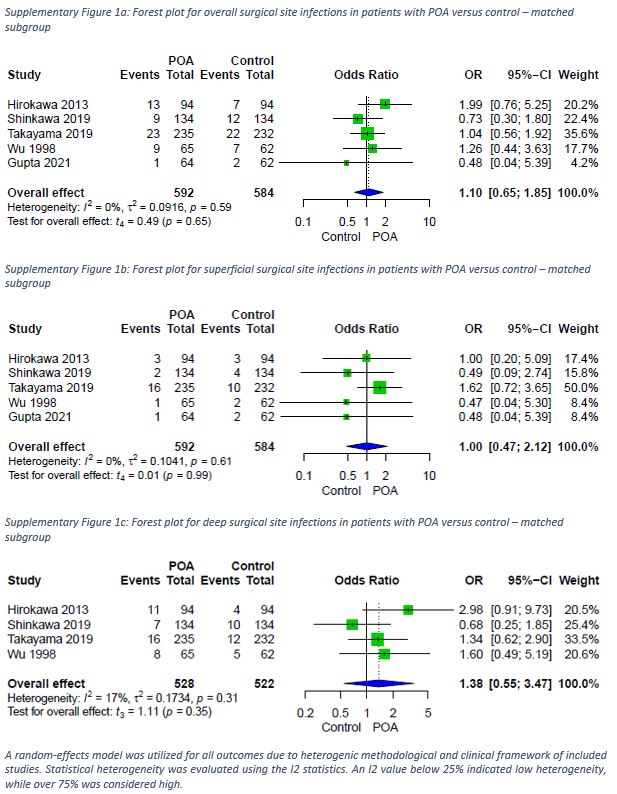

Supplement: Supplementary file 1 [file antibiotics-11-00649-s001.zip › Supplementary Figure S1.JPG]

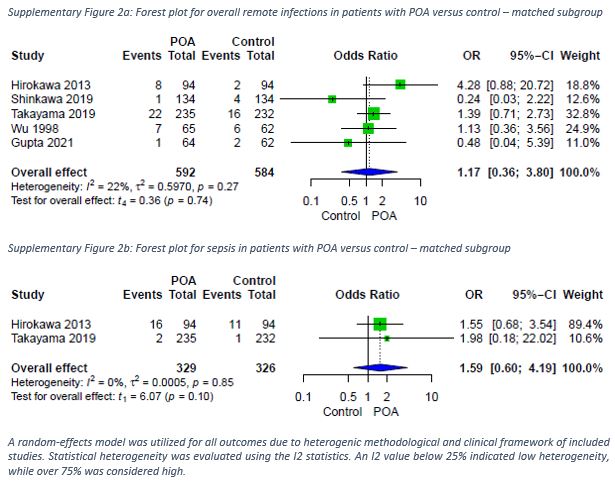

Supplement: Supplementary file 1 [file antibiotics-11-00649-s001.zip › Supplementary Figure S2.JPG]

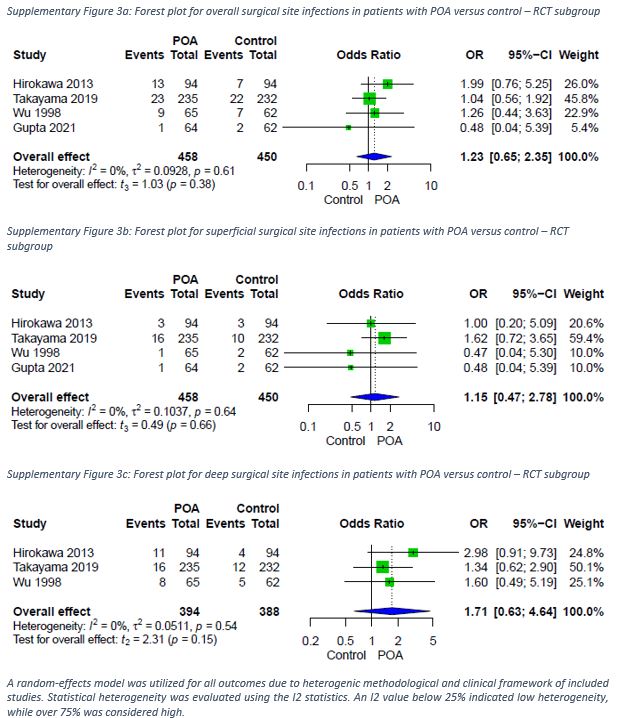

Supplement: Supplementary file 1 [file antibiotics-11-00649-s001.zip › Supplementary Figure S3.JPG]

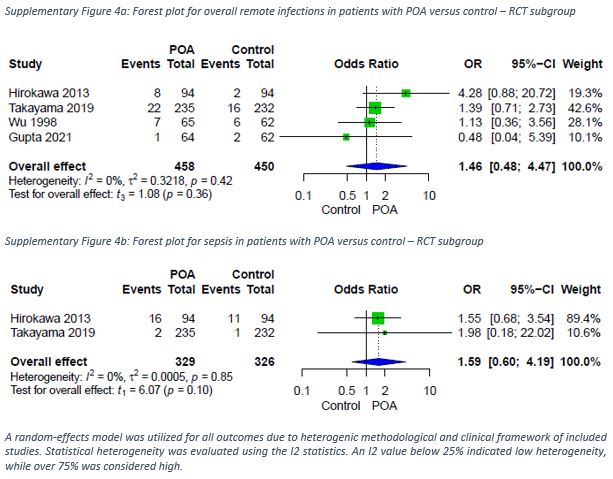

Supplement: Supplementary file 1 [file antibiotics-11-00649-s001.zip › Supplementary Figure S4.JPG]
